# CO_2_ Emission Increases with Damage Severity in Moso Bamboo Forests Following a Winter Storm in Southern China

**DOI:** 10.1038/srep30351

**Published:** 2016-07-29

**Authors:** Sheng Liu, Hangmei Xu, Jiuming Ding, Han Y. H. Chen, Jiashe Wang, Zikun Xu, Honghua Ruan, Yuwei Chen

**Affiliations:** 1 Laboratory of Poyang Lake and Wetland Ecosystem Research, Nanjing Institute of Geography and Limnology, Chinese Academy of Sciences, Nanjing 210008, PR China; 2 Graduate University of Chinese Academy of Sciences, Beijing 100049, PR China; 3 Co-Innovation Center for Sustainable Forestry in Southern China, College of Biology and the Environment, Nanjing Forestry University, Nanjing, 210037, China; 4 Lianyungang Technical College, Lianyungang, Jiangsu Province 222006, China; 5 Faculty of Natural Resources Management, Lakehead University, 955 Oliver Road, Thunder Bay, ON, P7B 5E1 Canada; 6 Administrative Bureau of Wuyishan National Nature Reserve, Wuyishan, Fujian 354300, China

## Abstract

Despite the prevalence of disturbances in forests, the effects of disturbances on soil carbon processes are not fully understood. We examined the influences of a winter storm on soil respiration and labile soil organic carbon (SOC) of a Moso Bamboo (*Phyllostachys heterocycle*) plantation in the Wuyi Mountains in Southern China from May 2008 to May 2009. We sampled stands that were damaged at heavy, moderate, and light levels, which yielded aboveground biomass inputs to the soil at 22.12 ± 0.73 (mean ± 1 s.e.m.), 10.40 ± 1.09, and 5.95 ± 0.73 Mg per hectare, respectively. We found that soil respiration rate and annual cumulative CO_2_ emissions were significantly higher in heavily damaged sites than moderately and lightly damaged sites. Soil temperature was the most important environmental factor affecting soil respiration rate across all studied stands. However, soil respiration sensitivity to temperature (Q_10_) decreased in heavily damaged sites. Microbial biomass carbon and its proportion to total SOC increased with damage intensity. Soil respiration rate was positively correlated to microbial biomass carbon and soil moisture. Our results indicated that the increase of soil respiration following canopy disturbance from winter storm resulted from increased microbial biomass carbon, soil moisture, and temperature.

Since forests play a critical role in the global carbon cycle, it is important to elucidate how common disturbances in forests may translate to strong impacts on their carbon balance. Disturbances alter forest structures and affect forest carbon dynamics. Initially, disturbances transfer carbon from the live biomass pool to plant detritus, which becomes available for decomposition. Secondly, disturbances modify the physical and chemical characteristics of forest soils, as well as microclimatic environments; hence, they may interact with increased detritus to affect carbon dynamics^1^. Previous studies have focused on the effects of disturbances on the mechanical damage characteristics of individual trees, soil quality, and the carbon stocks of ecosystems^2^,^3^,^4^. However, how soil respiration is influenced by disturbances in forest ecosystems remains poorly understood.

Soil respiration is the second largest flux (98 ± 12 Pg C yr^−1^) in the global carbon cycle, following photosynthesis^5^. Soil respiration is driven primarily by the microbial decomposition of soil organic matter and root respiration^6^, i.e., heterotrophic respiration and autotrophic respiration, respectively. Abiotic and biotic factors that mediate microbial growth and activity, as well as the allocation of C to plant roots, exert a considerable influence over soil respiration rates^7^. Abiotic factors, such as soil temperature, were found to have potent exponential relationships with soil respiration rates across various ecosystems^8^,^9^,^10^. Consequently, soil respiration sensitivity to temperature (Q_10_) has been widely used to evaluate the response of soil respiration to global warming^11^. Soil respiration rates and sensitivity to temperature may differ with different disturbance regimes including qualitative differences such as increased, decreased, or no responses of soil respiration rates and the sensitivity of soil respiration to temperature to disturbances^12^,^13^,^14^,^15^,^16^,^17^.

Further, soil moisture is an important factor in impacting soil respiration rates. However, the relationship between soil moisture and the soil respiration rate is typically not as strong as soil temperature^16^,^18^. Additionally, substrate availability may affect soil respiration rates^6^,^19^. Organic residue has been reported to markedly affect soil respiration^20^,^21^. Previous research has indicated that increased labile soil organic matter from the decomposition of organic residues may enhance microbial activity, and generally increases soil respiration^22^,^23^.

Severe winter storms occur with a mean interval of ~50 to 100 years in Chinese subtropical forests^24^. In January 2008, an unprecedented winter storm developed over Southern China, a prominent region for China’s terrestrial carbon storage^25^. This winter storm damaged 20.86 million ha of natural forests and plantations, which accounted for one tenth of the forests in Southern China^26^. The storm caused serious damage to 2.43 million ha of a total of 4.99 million ha of bamboo forests in China^27^,^28^. As a result, the winter storm produced abundant detrital materials (bamboo leaves, branches, and stems), and the damage severity varied greatly with topographical variables and stand attributes^2^. Previous studies have reported that soil temperature and moisture change following the canopy damage by similar storms^1^, and labile soil organic carbon increases due to increased litter input^29^. Therefore, it is expected that soil respiration would increase. However, what are the impacts of winter storms on bamboo forest carbon processes, and the mechanisms that are subsequently involved? At present, little data is available as relates to changes in the soil carbon process, and how winter storms might consequently affect carbon dynamics.

In this study, we conducted field experiments to examine how soil respiration rates and labile SOC were altered with damage intensity during the 2008 winter storm in a Moso Bamboo plantation. We hypothesized that soil respiration and labile soil organic carbon (SOC) increases following the winter storm, as plant litter and the generation of coarse woody debris increase with the extent of canopy damage, and increased soil temperatures and moisture facilitate SOC decomposition.

## Results

### Soil temperature and soil moisture content

**Figure 1:**
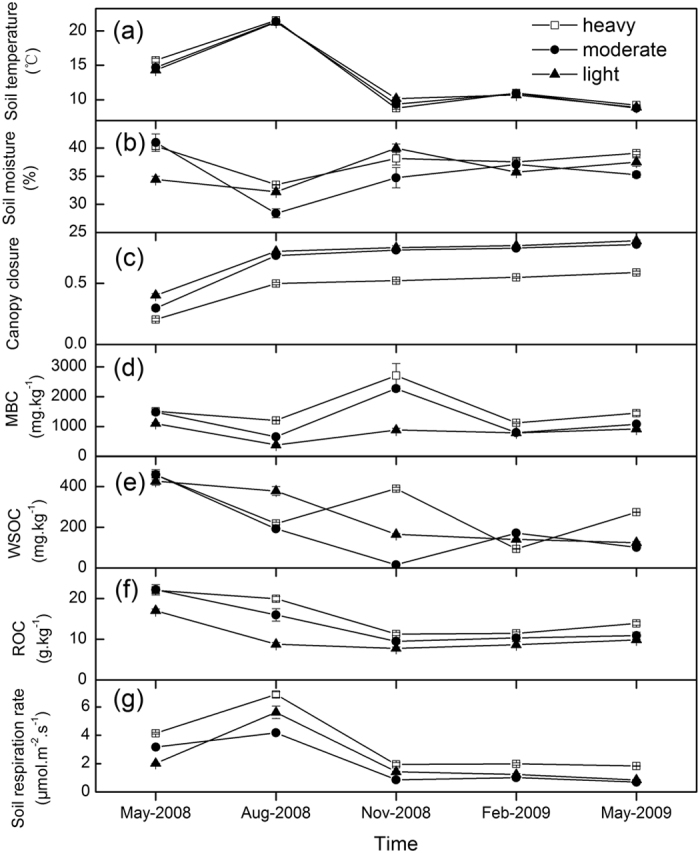
Temporal variations of soil temperature at 5-cm depth (**a**), soil moisture content in the 0–10-cm soil layer (**b**), canopy closure (**c**), microbial biomass carbon (MBC) in the 0–10 cm soil layer (**d**), water-soluble organic carbon (WSOC) in the 0–10 cm soil layer (**e**), readily oxidizable carbon (ROC) in the 0–10 cm soil layer (**f**), and soil respiration rate (**g**) among damage levels during the experimental period. Error bars are standard error (*n* = 3).

The chemical composition and physical characteristics of the soil did not differ under variable damage levels (Table 1). The seasonal pattern of soil temperatures were strongly varied among three damage levels with significantly higher soil temperatures the heavily damaged level than other levels in May 2008 (Fig. 1a; Table 2). Variations in soil moisture (0–10 cm) across measurement periods followed a pattern that was opposite to that of soil temperature (Fig. 1b; Table 2). The annual average soil moisture was higher for the heavily damaged level in contrast to the moderately and lightly damaged levels (Table 2). The annual mean canopy closure decreased significantly with damage intensity (Fig. 1c; Table 2), whereas the canopy closure at different damage levels gradually increased over time following the winter storm (Fig. 1c).

### Labile SOC

Temporal variations of microbial biomass carbon (MBC), water-soluble organic carbon (WSOC), and readily oxidizable carbon (ROC) were observed under different damage levels in the bamboo forests (Fig. 1d–f; Table 2). The annual mean MBC, WSOC, and ROC at the heavily damaged level were significantly higher than at the moderately and lightly damaged levels, and there was also a significant difference between the moderately and lightly damaged levels (Table 2). MBC were the highest during November 2008 for heavily, moderately, and lightly damaged levels (Fig. 1d). In May 2008, the highest WSOC values were shown for heavily, moderately, and lightly damaged levels (Fig. 1e). Also, in May 2008, heavily, moderately, and lightly damaged levels exhibited the highest ROC (Fig. 1f). The percentage of labile SOC to TOC revealed significant variations at the same soil depth over different damage levels, except at 25–40 cm, and there were no significant differences among soil depths for the same damage levels (Table 3).

### Soil respiration rates

**Figure 2:**
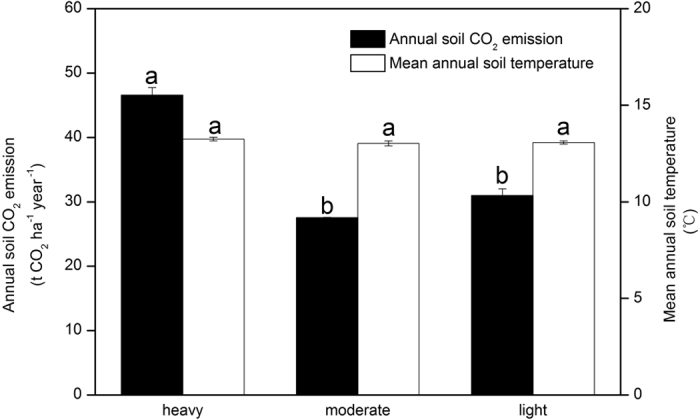
Mean annual soil temperature and annual soil CO_2_ emission among damage levels. Error bars are standard error (*n* = 3). Different letters over the bars indicate statistically significant differences at the 0.05 level of significance for damage levels.

Soil respiration rates differed with the damage level and sampling dates (Table 2). Variations in soil respiration rates (Fig. 1g) followed a seasonal pattern similar to that of soil temperatures (Fig. 1a). Soil respiration rates were the lowest during May 2009 and the highest during August 2008. The soil respiration rate for the heavily damaged level was significantly higher than the moderately and lightly damaged levels, and there was no significant difference between the moderately and lightly damaged levels (Table 2). The annual mean soil respiration rates for the heavily, moderately, and lightly damaged levels were 3.36, 1.98, and 2.23 μmol m^−2^ s^−1^, respectively. The annual cumulative soil CO_2_ emissions for the heavily damaged level was 46.6 ± 1.2 (mean ± 1 s.e.m.) t CO_2_ ha^−1^ yr^−1^, which was significantly higher than the moderately damaged level (27.5 ± 0.1 t CO_2_ ha^−1^ yr^−1^) and the lightly damaged level (31.0 ± 1.1 t CO_2_ ha^−1^ yr^−1^). There was no significant difference in the annual cumulative soil CO_2_ emissions between the moderately and lightly damaged levels (Fig. 2).

### Relationships between soil respiration rate and soil properties

Pearson’s correlation analysis showed that the soil respiration rate was significantly positively correlated with the soil temperature (*P* < 0.01) (Table 4). The Q_10_ values were significantly lower for the heavily damaged level in comparison to the moderately and lightly damaged levels at corresponding soil depths, and there was no significant difference between the moderately and lightly damaged levels (Table 5). The Q_10_ values calculated for soil temperatures measured at 5-, 10-, and 15-cm depths did not differ in the moderately and lightly damaged levels, but the value was significantly lower when calculated for the soil temperatures measured at a 5-cm depth, than at the other depths for the heavily damaged level (Table 5). The bivariate relationship showed that soil respiration rate was significantly negatively correlated with the soil moisture (*P* < 0.05) (Table 4). A significant positive relationship between the ROC and the soil respiration rate (*P* < 0.01) was found (Table 4). A noteworthy positive correlation was observed between the soil respiration rate and the WSOC (*P* < 0.01) (Table 4). No significant correlation was found between the soil respiration rate and the MBC (Table 4).

The multiple linear regression analysis, based on Akaike information criterion selection, indicated that soil temperature, soil moisture content, and microbial biomass carbon were significantly related to soil respiration (*F* = 123.592, *r* = 0.949, soil respiration = −7.256 + 0.427 × soil temperature + 9.872 × soil moisture content +0.0005 × microbial biomass carbon). The partial R^2^s were 0.843, 0.036, and 0.0216 for soil temperature, soil moisture, and microbial biomass carbon, respectively, with all predictor being significant (*P* < 0.01). The standard partial regression coefficient of soil temperature was 5.74 and 6.70 times greater than that of soil moisture and microbial biomass carbon, respectively, indicating that soil temperature was the most important variable that influenced soil respiration.

## Discussion

The soil water-soluble organic carbon, microbial biomass carbon, and readily oxidizable carbon increased with the damage level following the winter storm, which supported our hypothesis. The biomass loss initiated by the winter storm increased with damage intensity, which led to differences in the spatial distribution and quantity of litter and coarse woody debris for different damage levels. This finding was consistent with Wang *et al*.^30^, who demonstrated that the surface application of bamboo leaves increased the water-soluble organic carbon and microbial biomass carbon in the soil of an intensively managed Chinese chestnut plantation. The addition of fresh bamboo leaves may provide humus and labile organic C pools, which are beneficial for the growth of soil resident microorganisms^31^. Additionally, Bhattacharyya *et al*.^32^ showed that the combined application of rice straw and green manure was more effective in increasing microbial biomass carbon, water-soluble organic carbon, and readily oxidizable carbon; a similar conclusion was found by Jiang *et al*.^33^, and Li *et al*.^21^. Furthermore, higher percentages of labile soil organic carbon to total soil carbon concentrations at the heavily damaged level, in comparison to the moderately and lightly damaged levels, showed that higher labile soil organic carbon was derived from the decomposition of litter and coarse woody debris, which was caused by the winter storm.

Our study indicated that the emission CO_2_ from the soil increased with damage level. The emission of CO_2_ from Moso Bamboo plantation soil under various disturbance models exhibited significant differences. Intensive management practices, such as regular tillage, weeding, high stand density, and fertilizer application, have increasingly been employed by farmers to improve the growth rates of bamboo forests toward the maximization of economic return^34^. The total annual CO_2_ emission from soil tends to increase with management intensity in bamboo forests^13^. Zhang *et al*.^14^ found that converting a paddy field to bamboo stands significantly reduced soil CO_2_ emissions, the paddy field and bamboo stands emitted 45.4 and 34.7 t CO_2_ ha^−1^ yr^−1^, respectively, of cumulative CO_2_ emissions. In our study, the winter storm produced a significant degree of carbon transition, from living biomass to dead biomass. Moreover, when considering the death of bamboo roots and rhizome, caused by uprooted and snapped trees, additional carbon would be involved in this shift^2^. Although the carbon contained within non-living biomass is not immediately respired to the ambient atmosphere, the detritus pulse largely represents committed future CO_2_ emissions^35^. If a warming climate initiates more extreme events with greater storm intensity, elevated tree mortality will generate additional litterfall and coarse woody debris, which results in higher ecosystem respiration, and a potentially important positive feedback with elevated atmospheric CO_2_^36^. Immediately following winter storms, Moso Bamboo forests may serve as sources of carbon to the ambient atmosphere until the photosynthetic uptake from the regrowth of vegetation becomes higher than the soil respiration from decomposing non-living biomass. Therefore, the input of litterfall and coarse woody debris caused by winter storms is a dominant factor in increasing short-term soil CO_2_ emissions in Moso Bamboo forests.

The conversion of live biomass to detritus was the core translational factor for this winter storm. The quantity of aboveground dead biomass of 5.95 to 22.12 Mg per hectare (averaging 12.83 ± 4.80 Mg per hectare) at different damage levels exceeded the annual accumulated aboveground litterfall in undisturbed bamboo plantations to a considerable degree. It has been previously reported that the typical annual litter biomass of undisturbed Moso Bamboo forests was 1.66 Mg per hectare in on-year, and 1.93 Mg per hectare in off-year in the similar climate region, respectively^27^. An average of 16.42 ± 7.09 Mg per hectare of dead dry biomass was generated in the central Moso Bamboo growth areas in Fenyi, Jiangxi Province, China after the 2008 winter storm, as reported by Zhou *et al*.^2^. Thus, a single climatic event might produce quantities of dead biomass that are approximately two- to four fold higher than the nominal annual production of litterfall under a similar climate^2^.

Organic residues have been reported to increase soil respiration^17^,^29^,^31^,^32^. Consistent with these results, the winter storm produced abundant detrital materials (bamboo leaves, branches, and stems), which increased the soil respiration rate considerably, which supported the hypothesis of our study. Increased dead biomass leads to additional labile soil organic matter within the surface soil, which acts to enhance microbial activity toward generally increasing soil respiration^20^,^30^. This increase in soil respiration via an increase in litter input might be at least partially explained by associated increases in the microbial biomass carbon and decomposition rates^22^,^29^.

Previous studies have indicated that soil respiration rates are greatly regulated by temperature in forest^15^,^30^,^37^,^38^, and grassland soils^10^. Increasing temperatures improve microbial activity, which contributes significantly to soil respiration, and exponentially increases CO_2_ emissions. Our study supported this relationship regardless of the damage level, with soil temperature accounting for 84% of the variations in soil respiration, which suggested that soil temperature was the primary driving force in the variation of soil respiration. The Q_10_ value of soil respiration varied from 2.95 to 4.91 across different damage levels, with the average of 4.05 higher than the global median value of 2.4 (1.3 to 3.3)^5^. The higher Q_10_ value in our study might have primarily resulted from the characteristics of root respiration, soil organic carbon availability, or microbial activities in Moso Bamboo forests, which were seriously impacted by the winter storm. The lower Q_10_ value of soil respiration for the heavily damaged level, in comparison to the moderately and lightly damaged levels, demonstrated that the sensitivity of soil respiration to temperature decreased at the higher damage levels. Liu *et al*.^13^ indicated that management practices, such as fertilizer application, soil tillage, and weeding, may have the effect of reducing the temperature sensitivity of soil respiration. However, several studies have revealed that intensive anthropogenic management practices (e.g., cultivation, fertilization, and irrigation) might promote the decomposition of soil organic matter, enhance microbial activity, and further increase the temperature sensitivity of soil respiration^12^,^30^,^37^. The Q_10_ value could be affected by a number of factors, including calculation method, temperature, moisture range, substrate quality, and microbial population^39^,^40^. The lower Q_10_ value in the heavily damaged level would be beneficial for decreasing soil CO_2_ emissions and increasing soil C storage, in contrast to other damage levels.

In our study, Q_10_ values did not differ with the depth of soil temperature measurement in moderately and lightly damaged levels, except when measured at the 5-cm depth for the heavily damaged level. The depths at which the soil temperature is measured may affect Q_10_ values^41^,^42^. Generally, Q_10_ values increase with the soil depth. The temperature measurement depth for developing the soil temperature and soil CO_2_ emission relationship is contingent on the land-use type and associated management practices^30^. Hence, this factor should be considered when using Q_10_ as an element of a model to predict the response of soil respiration to global warming, particularly at the landscape scale^42^.

There was a significantly positive relationship between soil respiration and soil moisture in our study, which was not as strong as that between soil respiration and soil temperature. Previous studies have indicated that soil moisture may have little effect on soil respiration in the subtropical regions of China, where rainfall is plentiful and prolonged drought is rare^37^,^43^, and soil moisture was not a limiting factor for soil CO_2_ production in this subtropical site^44^, since variations in soil moisture were relatively small. However, Tang *et al*.^45^ reported that CO_2_ emissions from the soil were positively correlated with soil moisture in pine, mixed, and broadleaf forests in a subtropical area. It has been suggested that the effects of soil moisture on soil respiration are dependent on the land-use type, management practices, and climate conditions^13^,^18^,^37^.

Labile SOC has been reported to be closely correlated with soil respiration^46^,^47^. Here, we discovered that soil respiration was significantly positively correlated with microbial biomass carbon. The growth of soil microorganisms was indicated by the increase of microbial biomass carbon via the application of organic residue (a C source)^22^. In our study, we also found that the input of litter and coarse woody debris caused by the winter storm increased the microbial biomass carbon content, which explained the higher soil respiration rate. No significant relationship was found between soil respiration and water-soluble organic carbon, and readily oxidizable carbon in our study. A possible explanation might be the strong dependence of soil respiration on temperature, which weakened the relationship between the water-soluble organic carbon, readily oxidizable carbon, and soil respiration^13^.

The winter storm of 2008 generated a high volume of litter and coarse woody debris in bamboo forests, and increased soil respiration and labile SOC. Soil temperature accounted for 84% of the variations in soil respiration, where soil respiration sensitivity to temperature (Q_10_) decreased at the higher damage intensity. Soil moisture and microbial biomass carbon were positively correlated to soil respiration rate. Our study suggested that increased litter and coarse woody debris, as well as the altered microclimate in bamboo forests following the winter storm, might increase soil respiration and labile SOC. The winter storm had a significant influence on carbon flux in the Moso Bamboo forest ecosystems. We noted that there was a large variation in Bamboo plantation ages, topography, and regional climate, as well as the range of disturbance intensity. Future efforts are required to fully understand the spatial variations in soil respiration and long-term temporal changes following extreme climate events such as the winter storm of 2008 in southern China.

## Methods

### Study area

This study was carried out at the Wuyi National Nature Reserve in the Northern Fujian Province, a 56,527 ha forest in southeastern China (117°27′~117°51′E, 27°33′~27°54′N). The annual mean temperature was 15.0 °C, with a relatively humidity of 83.5%, and annual mean precipitation of 2,000 mm. The experiment site was located in the Moso Bamboo forest at 960 m above sea level, with a northeast aspect and slope of 25°. The mean stand age was two years old, the average diameter at breast height (DBH, 1.3 m above root collar) was 12 cm, the median height was 16.0 m, and the bamboo density was 2,000 individuals per ha. The dominant herbs species included *Isachne globosa*, *Boenninghausenia albiflora*, *Viola verecunda*, *Ophiopogon bodinieri*, and *Carex* spp. The Moso Bamboo forest was harvested when the culms were older than six years, and was otherwise unmanaged.

### Stand selection and measurements

Field observations were conducted to identify and locate the Moso Bamboo plantations that were damaged at different intensities following the storm. Nine stands were randomly selected to represent heavy, moderate, and light damage levels; each with three replicated stands, across the study area. Within each stand, a 100 m^2^ (10 × 10 m) plot was established in May 2008. The DBH, height, and age of each bamboo tree were measured and recorded within each plot. During the selection of the sites, particular attention was given to ensuring the similarity of the following permanent site conditions: soil type and texture, parent material, climate, drainage, and topography.

We quantified the damaged levels in each plot by calculating the percentage of bamboo culms that were snapped or uprooted by the storm. On average, the heavily damaged plots had 43.68% ± 2.12% (mean ± 1 s.e.m.) of its bamboo culms damaged, whereas moderately damaged plots had 21.81% ± 0.44%, and light had 10.27% ± 0.76%, respectively.

### Measurement of canopy closure and winter storm induced biomass loss

The canopy closure of each plot was measured by a WinScanopy canopy analyzer. The dead biomass for each plot was the sum of the snapped and uprooted individuals as they died off within a few months following the ice storm^2^. We employed published biomass allometric equations developed in this study area^48^, which utilized DBH and height as predictors for biomass, to calculate individual bamboo biomass.

### Soil respiration measurements

In each plot, one polyvinyl chloride collar (5 cm in depth and 10 cm in diameter) was inserted into the soil to measure soil respiration. Nine collars were installed one month prior to the initial sample collection. Before the soil respiration measurement, the aboveground components of all living plants within the collar were carefully removed by hand. The soil respiration rate was measured using a LI-6400-09 soil chamber, which was connected to a portable infrared gas analyzer (LI-6400, LI-COR, Inc., Lincoln, NE) that was positioned above the collar. At each sampling date, the soil respiration rate was measured three times for each plot, and the three values were averaged to arrive at one value per plot, per date. All measurements were made between 9:00 and 11:00 am local time, since soil respiration rates during this period are close to the daily mean in this subtropical region^45^. However, there are limitations to this approach^30^ and the annual soil CO_2_ emission calculations (below) should be considered as approximations of the actual annual rates, and are used in this paper for comparing the differences among damaged levels. At the time of gas sampling, the soil temperature was measured with a LI-6400 thermal detector at soil depths of 5-, 10-, and 15-cm, whereas soil moisture content at soil depths of 0–10, 10–25, and 25–40 cm was determined as the difference between the field moisture and dried soil weight (24 h oven dry at 105 °C). The field measurements were carried out every three months, from May 2008 to May 2009. We calculated the annual soil CO_2_ emission using the following equation^14^:


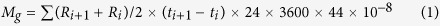


where *M*_*g*_ is annual soil CO_2_ emission (t CO_2_ ha^−1^ year^−1^), *R* is the soil respiration rate (μmol CO_2_ m^−2^ s^−1^) determined at each sampling time, *i* is the sampling number, and *t* is the sampling time, multiplied by 24 and 3600, to convert time from seconds to days and by (44/1 0000 0000) to convert CO_2_ from micromoles to tons.

We used a Q_10_ model to analyze the relationship between soil respiration and soil temperature. Q_10_ is the sensitivity of soil respiration to temperature when the temperature rises 10 °C:


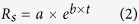



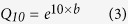


where *R*_*s*_is the soil respiration rate (μmol CO_2_ m^−2^ s^−1^), *t* is soil temperature (°C), *a* and *b* are the fitting constants, and *e* is the exponential base.

### Analyses of soil chemical and physical properties

Soil samples were randomly collected (0–10 cm, 10–25 cm, and 25–40 cm depths) from each of the plots in May 2008, using a Ø = 2 cm soil corer. Twenty soil cores were extracted from each plot at each soil depth, and then pooled together as a composite sample for laboratory analysis. Samples were immediately sieved (<2 mm) to remove any visible soil fauna, stones, and fine roots, and then divided into two portions. One was kept in the refrigerator at 4 °C, prior to the analysis of MBC and WSOC, and the other was air-dried for readily oxidizable carbon (ROC) analysis. The total soil carbon concentration (TOC) and total nitrogen concentration (TN) were determined through combustion with an elemental analyzer (Model CNS, Elementar Analysen Systeme GmbH, Germany). The soil pH was measured in a 1:2.5 soil-water suspension using a glass electrode. Soil samples for the analysis of soil bulk density were extracted via a steel cylinder of 100-cm^3^ volume (5 cm in diameter, 5 cm in height).

### Measurement of labile SOC

The MBC was measured through a chloroform fumigation-extraction method^49^, and the MBC concentration in the extracted solutions was measured with a TOC Analyzer (Shimadzu, TOC-Vcph, Japan). WSOC was extracted from 20 g of fresh soil with the addition of 40 ml of deionized water, and the mixture was agitated for 0.5 h at 250 rpm at 25 °C, and centrifuged for 10 min. at 15,000 rpm. Subsequently, the supernatant liquid was filtered through a 0.45 mm membrane^33^. The WSOC in the extracts was measured by a Shimadzu TOC Analyzer. ROC was measured using a KMnO_4_ oxidation procedure and a spectrophotometer^50^.

### Statistical analysis

We used one-way ANOVA to test the differences in annual soil CO_2_ emission and Q_10_ among damage levels. Repeated measures of ANOVA were used to identify the effects of damage levels on soil respiration rates, labile SOC, soil temperature, soil moisture content, and canopy closure. We used Pearson correlation analysis to examine the associations among soil respiration rate, labile SOC, soil temperature, soil moisture content, and canopy closure. We employed multiple linear regression to identify and quantify the relationships between the soil respiration rate and labile SOC, soil temperature, soil moisture content, and canopy closure. We used Akaike information criterion (AIC) to select the ‘best’ model, i.e., the model with the lowest AIC, for interpretation. All statistical analyses were conducted using SPSS 15.0 software (SPSS Institute Inc., Chicago, IL, USA).

## Additional Information

**How to cite this article**: Liu, S. *et al*. CO_2_ Emission Increases with Damage Severity in Moso Bamboo Forests Following a Winter Storm in Southern China. *Sci. Rep.* **6**, 30351; doi: 10.1038/srep30351 (2016).

## Figures and Tables

**Table 1:** Comparison of soil chemical and physical properties of different damage levels in the bamboo forest.

Damaged level	Soil depth (cm)	pH	TOC (g.kg^−1^)	TN (g.kg^−1^)	C/N	Bulk density (g.cm^−3^)
Heavy	0–10	5.65 ± 0.26a	45.05 ± 3.61a	3.88 ± 0.44a	11.84 ± 1.40a	0.85 ± 0.06a
	10–25	5.55 ± 0.08a	27.03 ± 2.22a	2.98 ± 0.20a	9.24 ± 1.44a	0.95 ± 0.01a
	25–40	5.49 ± 0.17a	21.09 ± 3.05a	2.18 ± 0.27a	10.24 ± 2.69a	0.97 ± 0.03a
Moderate	0–10	4.76 ± 0.23a	44.03 ± 1.50a	3.99 ± 0.14a	11.07 ± 0.51a	0.86 ± 0.02a
	10–25	5.07 ± 0.19a	28.65 ± 3.46a	2.87 ± 0.30a	9.96 ± 0.36a	0.92 ± 0.03a
	25–40	4.97 ± 0.13a	19.98 ± 2.56a	1.99 ± 0.24a	10.01 ± 0.08a	1.11 ± 0.05a
Light	0–10	4.95 ± 0.21a	43.31 ± 1.70a	3.39 ± 0.25a	12.98 ± 1.31a	0.81 ± 0.01a
	10–25	5.50 ± 0.23a	28.65 ± 0.89a	2.74 ± 0.03a	10.45 ± 0.42a	0.93 ± 0.03a
	25–40	5.32 ± 0.09a	18.26 ± 2.59a	1.87 ± 0.17a	9.72 ± 0.65a	1.03 ± 0.01a

Note: Values are mean ± 1 s.e.m., Means within a column with different letters are significantly different among damage levels at the same depth (*P* < 0.05). TOC, total soil carbon concentration; TN, total nitrogen concentration; C/N, carbon: nitrogen ratio.

**Table 2:** Statistically significant differences of soil temperature, soil moisture content, canopy closure, soil respiration rate, MBC, WSOC, and ROC, based on repeated ANOVA measures.

Variable	Damaged level	Time	Damaged level × Time
Soil temperature	0.338	<0.001	<0.001
Soil moisture content	0.035	<0.001	<0.001
Canopy closure	<0.001	<0.001	<0.001
Soil respiration rate	<0.001	<0.001	<0.001
MBC	<0.001	<0.001	<0.001
WSOC	<0.001	<0.001	<0.001
ROC	<0.001	<0.001	<0.001

Note: MBC, microbial biomass carbon; WSOC, water-soluble organic carbon; ROC, readily oxidizable carbon. Values in table are *P*.

**Table 3:** Variations of the percentages of labile SOC to TOC at different soil depths for different damage levels in the bamboo forests.

Damaged level	Soil depth (cm)	MBC/TOC (%)	WSOC/TOC (%)	ROC/TOC (%)
Heavy	0–10	3.60 ± 0.36Aa	0.65 ± 0.05Aa	35.50 ± 3.61Aa
	10–25	4.34 ± 0.31Aa	0.68 ± 0.06Aa	35.20 ± 2.83Aa
	25–40	3.27 ± 0.49Aa	0.69 ± 0.09Aa	31.99 ± 4.01Aa
Moderate	0–10	2.86 ± 0.07Aa	0.43 ± 0.02Ab	31.31 ± 0.43Aab
	10–25	3.33 ± 0.44Aa	0.46 ± 0.06Ab	31.58 ± 3.23Aa
	25–40	2.58 ± 0.39Aa	0.46 ± 0.07Aa	30.98 ± 4.65Aa
Light	0–10	1.89 ± 0.09Ab	0.57 ± 0.03Aa	24.17 ± 1.43Ab
	10–25	2.20 ± 0.05Ab	0.60 ± 0.17Aab	21.49 ± 1.62Ab
	25–40	2.05 ± 0.27Aa	0.51 ± 0.07Aa	23.41 ± 3.35Aa

Note: Values are mean ± 1 s.e.m., Means within a column with different lowercase letters are significantly different from others at the same depth (*P* < 0.05). Means within a column with different capital letters are significantly different from others at the same damage level (*P* < 0.05). MBC, microbial biomass carbon; WSOC, water-soluble organic carbon; ROC, readily oxidizable carbon; TOC, total soil carbon concentration.

**Table 4:** Pearson correlation coefficients among soil temperature, soil moisture content, canopy closure, soil respiration rate, MBC, WSOC, and ROC.

Characteristic	Soil temperature	Soil moisture content	Canopy closure	Soil respiration rate	MBC	WSOC
Soil moisture content	−0.545^**^					
Canopy closure	−0.276	−0.351^*^				
Soil respiration rate	0.918^**^	−0.341^*^	−0.411^**^			
MBC	−0.394^**^	0.381^**^	−0.356^*^	−0.193		
WSOC	0.376^*^	0.244	−0.757^**^	0.452^**^	0.136	
ROC	0.486^**^	0.110	−0.841^**^	0.538^**^	0.145	0.600^**^

Note: MBC, microbial biomass carbon; WSOC, water-soluble organic carbon; ROC, readily oxidizable carbon.

*Probability < 0.05; **Probability < 0.01.

**Table 5:** Comparison of Q_10_ at different soil depths for different damage levels in the bamboo forest.

Damaged level	Soil depth	a	b	Q_10_
Heavy	5 cm	0.691 ± 0.028	0.108 ± 0.002	2.95 ± 0.058Aa
	10 cm	0.611 ± 0.029	0.117 ± 0.003	3.23 ± 0.081Ba
	15 cm	0.543 ± 0.033	0.121 ± 0.001	3.36 ± 0.040Ba
Moderate	5 cm	0.221 ± 0.009	0.148 ± 0.003	4.39 ± 0.144Ab
	10 cm	0.196 ± 0.011	0.155 ± 0.004	4.69 ± 0.185Ab
	15 cm	0.175 ± 0.010	0.159 ± 0.004	4.91 ± 0.196Ab
Light	5 cm	0.283 ± 0.021	0.141 ± 0.007	4.09 ± 0.271Ab
	10 cm	0.248 ± 0.016	0.147 ± 0.006	4.35 ± 0.266Ab
	15 cm	0.224 ± 0.018	0.151 ± 0.007	4.50 ± 0.306Ab

Note: Values are mean ± 1 s.e.m., Means within a column with different lowercase letters are significantly different from others at the same depth (*P* < 0.05). Means within a column with different capital letters are significantly different from others at the same damage level (*P* < 0.05).
